# The Prostate Cancer Cells Resistant to Docetaxel as in vitro Model for Discovering MicroRNAs Predictive of the Onset of Docetaxel Resistance

**DOI:** 10.3390/ijms18071512

**Published:** 2017-07-13

**Authors:** Lorenzo Bascetta, Arianna Oliviero, Romina D’Aurizio, Monica Evangelista, Alberto Mercatanti, Marco Pellegrini, Francesca Marrocolo, Sergio Bracarda, Milena Rizzo

**Affiliations:** 1 Non-Coding RNA Laboratory, Institute of Clinical Physiology (IFC), National Research Council (CNR), via G. Moruzzi 1, 56124 Pisa, Italy; lorenzobascetta@hotmail.it (L.B.); arianna90oli@hotmail.it (A.O.); m.evangelista@ifc.cnr.it (M.E.); alberto.mercatanti@ifc.cnr.it (A.M.); 2 Laboratory for Integrative System Medicine (LISM), Institute of Informatics and Telematics (IIT), National Research Council (CNR), via G. Moruzzi 1, 56124 Pisa, Italy; romina.daurizio@gmail.com (R.D.); marco.pellegrini@iit.cnr.it (M.P.); 3 Department of Oncology, San Donato Hospital, Azienda USL Toscana Sud-Est, via P. Nenni 20, 52100 Arezzo, Italy; francesca.marrocolo@uslsudest.toscana.it (F.M.); sergio.bracarda@uslsudest.toscana.it (S.B.); 4 Istituto Toscano Tumori (ITT), via T. Alderotti 26/N, 50139 Firenze, Italy

**Keywords:** prostate cancer cell lines, docetaxel resistance, circulating miRNAs, predictive biomarkers

## Abstract

On the grounds that miRNAs present in the blood of prostate cancer (PCa) patients are released in the growth medium by PCa cells, it is conceivable that PCa cells resistant to docetaxel (DCT) (DCT^R^) will release miRNAs that may be found in PCa patients under DCT therapy if resistant PCa cells appear. We isolated DCT^R^ clones respectively from 22Rv1 and DU-145 PCa cell lines and performed through next-generation sequencing (NGS) the miRNAs profiles of the released miRNAs. The analysis of the NGS data identified 105 and 1 miRNAs which were differentially released in the growth medium of the 22Rv1/DCT^R^ and DU-145/DCT^R^ clones, respectively. Using additional filters, we selected 12 and 1 miRNA more released by all 22Rv1/DCT^R^ and DU-145/DCT^R^ clones, respectively. Moreover, we showed that 6 of them were more represented in the growth medium of the DCT^R^ cells than the ones of DCT-treated cells. We speculated that they have the pre-requisite to be tested as predictive biomarkers of the DCT resistance in PCa patients under DCT therapy. We propose the utilization of clones resistant to a given drug as in vitro model to identify the differentially released miRNAs, which in perspective could be tested as predictive biomarkers of drug resistance in tumor patients under therapy.

## 1. Introduction

Prostate cancer (PCa) evolves into a condition known as castration-resistant prostate cancer (CRPC) when the androgen deprivation therapy fails. At this stage, docetaxel (DCT) represents the gold standard treatment [1]. Unfortunately, most patients develop resistance to the drug within a year from the beginning of the therapy, and the disease progresses [2]. The identification of biomarkers capable to predict in advance the onset of the DCT resistance could facilitate the shift towards alternative effective therapies, thus increasing the life expectancy of the patients.

MicroRNAs (miRNAs), and more recently circulating miRNAs (c-miRNA), have been proposed as molecules with a diagnostic and prognostic value for PCa [3,4] but only few studies explored c-miRNAs as predictive biomarker of DCT resistance [5,6].

We have already reported that PCa cells in vitro specifically released in the growth medium the same miRNAs found in the peripheral blood of PCa patients [7]. Therefore, it is conceivable that PCa cells resistant to DCT will release in the growth medium miRNAs that, if detected in the blood of PCa patients, could reveal the presence of DCT-resistant cells. To approach the problem, we isolated PCa cells resistant to DCT and used the next-generation sequencing (NGS) technology and bioinformatics tools to identify miRNAs differentially released in the growth medium by DCT-resistant cells (DCT^R^-miRNAs). We also discussed that DCT^R^-miRNAs may represent potential predictive biomarkers of the DCT resistance suitable to be tested in PCa patients under DCT therapy.

## 2. Results

### 2.1. Isolation of DCT^R^ Clones from 22Rv1 and DU-145 Prostate Cancer Cell Lines

**Figure 1:**
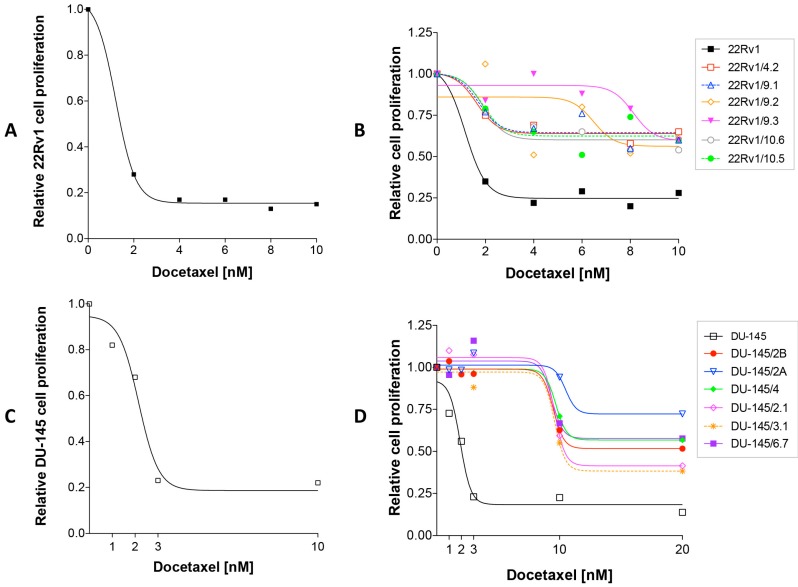
Effect of docetaxel (DCT) on DU-145 cell proliferation. Relative cell proliferation of 22Rv1 cells (**A**), 22Rv1/DCT^R^ clones (**B**), DU-145 cells (**C**) and DU-145/DCT^R^ clones (**D**) exposed to increasing concentrations of docetaxel for 72 h.

We exposed for three days either 22Rv1 or DU-145 PCa cells to increasing concentrations of DCT and observed a dose-dependent inhibition of cell proliferation which reached a plateau phase at 4 nM concentration and 3 nM, respectively (Figure 1A,C). We then grew continuously 22Rv1 cells and DU-145 cells respectively in 4 nM and 3 nM DCT-containing medium until we observed colonies presumably resistant to DCT. We isolated and expanded as clones 6 independent colonies from both 22Rv1 cells (22Rv1/DCT^R^ clones) and DU-145 cells (DU-145/DCT^R^ clones). Afterwards, we tested them for the DCT resistance. We treated cells with increasing DCT concentration and showed that 22Rv1/DCT^R^ and DU-145/DCT^R^ clones were more resistant to DCT with respect to parental cell lines (Figure 1B,D).

**Figure 2:**
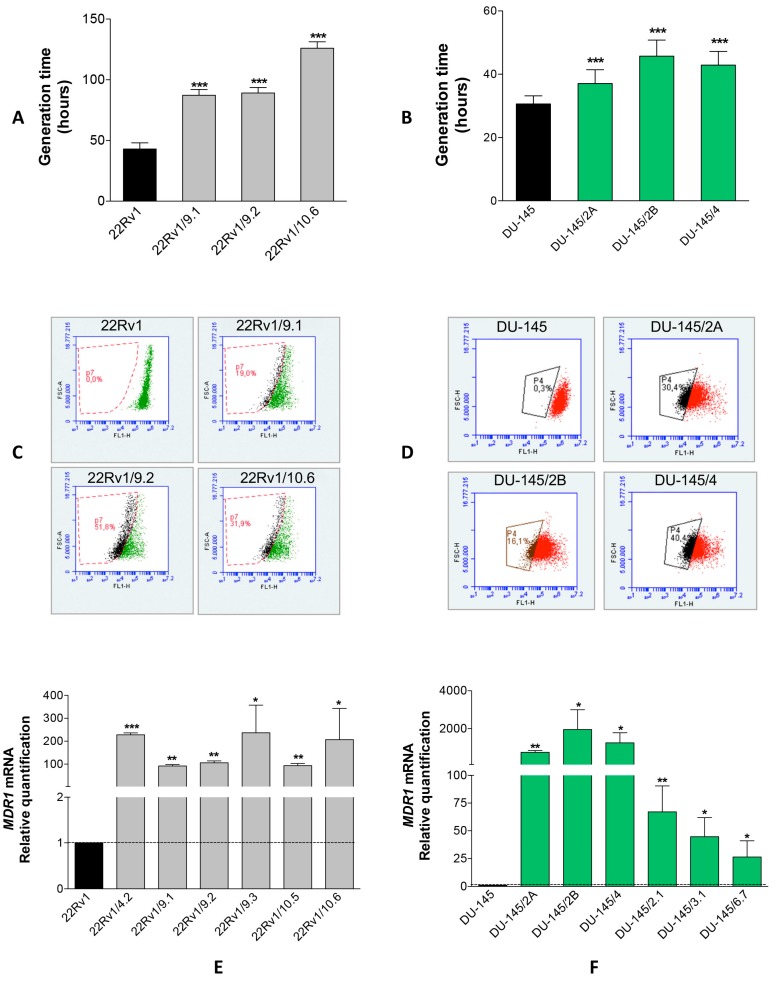
Characterization of DCT^R^ clones. Generation times (calculated dividing the hours in culture by the number of cell doublings) (**A**,**B**), percentage of cells which extruded Rh123 (**C**,**D**), *MDR1* mRNA relative quantification with qRT-PCR (**E**,**F**) in 22Rv1/DCT^R^ or DU-145/DCT^R^ clones with respect to parental cells (dotted line). Data were shown as mean ± SD from three independent experiments (* *p* < 0.05, ** *p* < 0.01, *** *p* < 0.001, unpaired *t*-test).

In addition, we showed that 22Rv1/DCT^R^ as well as DU-145/DCT^R^ clones presented expanded generation times (Figure 2A,B) and an acquired capability to extrude Rhodamine 123 (Figure 2C,D). These results, together with the upregulation of *MDR1* gene (Figure 2E,F), indicated that the drug export increase through the ABC transporters was at the basis of the DCT resistance in all clones. We tested also the expression of *BCL2* and *βIII tubulin* genes, whose upregulation is involved in the DCT resistance in PCa [8,9].

**Figure 3:**
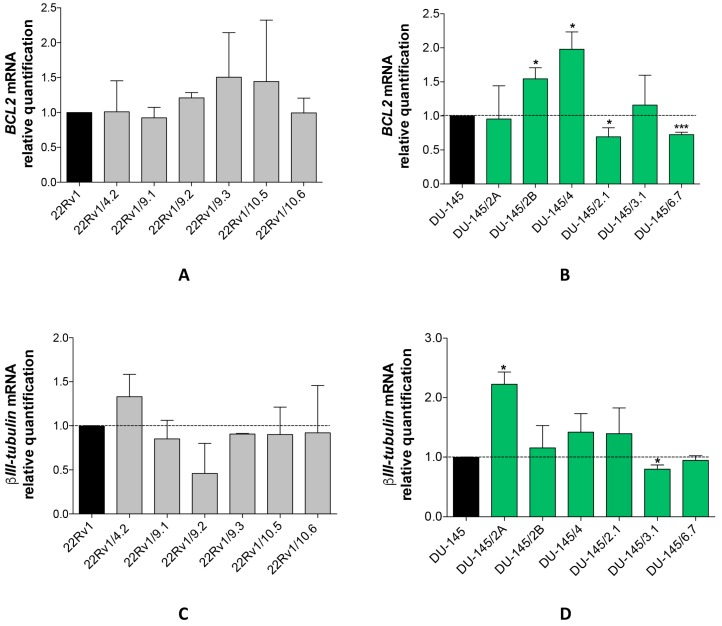
Characterization of DCT^R^ clones. *BCL2* (**A**,**B**) and *βIII tubulin* (**C**,**D**) mRNA relative quantification with qRT-PCR in 22Rv1/DCT^R^ or DU-145/DCT^R^ clones with respect to parental cells (dotted line). Data were shown as mean ± SD from three independent experiments (* *p* < 0.05, *** *p* < 0.001, unpaired *t*-test).

It is worth noting that while in 22Rv1/DCT^R^ clones the expression level of both *BCL2* (Figure 3A) and *βIII tubulin* (Figure 3B) genes did not change, these genes showed slight but significant variations in DU-145/DCT^R^ clones. In particular, *BCL2* was upregulated in DU-145/2B and DU-145/4 clones and downregulated in DU-145/2.1 and DU-145/6.7 clones (Figure 3B). In addition, *βIII tubulin* gene was upregulated in DU-145/2A clones and downregulated in DU-145/3.1 clones (Figure 3D).

### 2.2. Identification of miRNAs Differentially Released by DCT^R^ Clones

**Figure 4:**
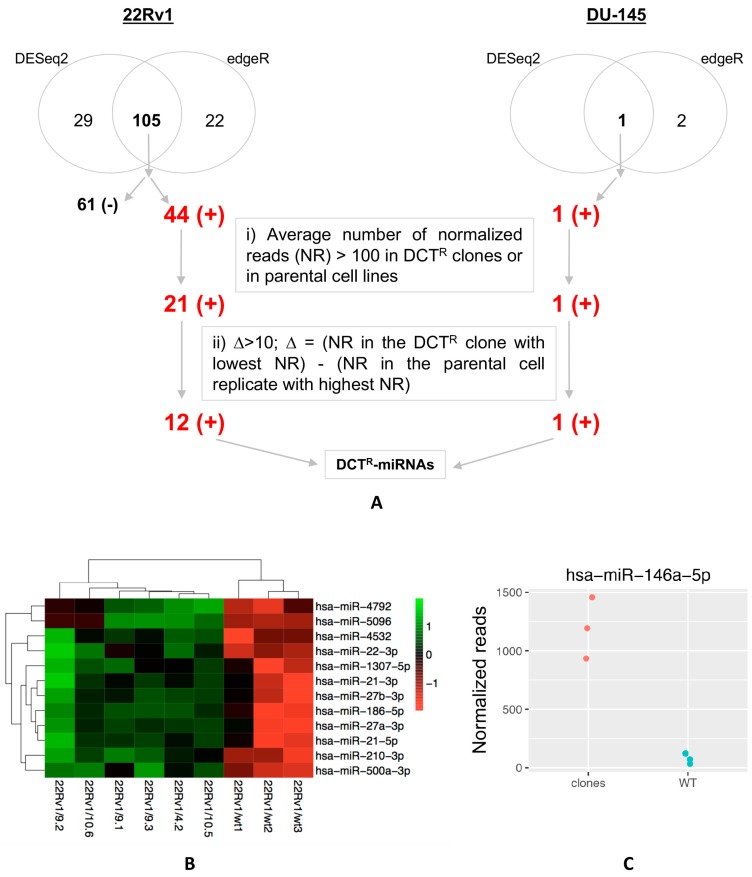
Extracellular miRNA profile of DCT^R^ clones. (**A**) Schematic representation of the selective criteria used to identify DCT^R^-miRNAs in both 22Rv1/DCT^R^ and DU-145/DCT^R^ clones (+, more released; −, retained/less released); (**B**) Heatmap representing the result of DCT^R^-miRNAs in 22Rv1/DCT^R^ clones and parental cells biological replicates (22Rv1WT1-3); (**C**) Normalized reads of miR-146a-5p in DU-145/DCT^R^ clones (red spots) and parental cells biological replicates (blue spots) of DU-145/2A, /2B and /4 clones (see Figure S2 for DU-145/2.1, /3.1 and /6.7 clones).

We collected the growth medium of each 22Rv1/DCT^R^ and DU-145/DCT^R^ clone as well as the one of parental cells and performed the miRNAs expression profile using next-generation sequencing (small RNA-seq). We conducted the differential analysis to identify, for each PCa cell line, the miRNAs that were differentially released with a statistical significance from all DCT^R^ clones with respect to the corresponding parental cells. The analysis was performed using two different methods, i.e., DESeq2 and edgeR. For DU-145/DCT^R^ clones, the small RNA-seq experiment was conducted in two different runs. To test whether the experiment could suffer a batch effect on distribution of detected miRNAs, we performed a principal component analysis and observed a significant bias (Figure S1). Therefore, for DU-145/DCT^R^ clones, we use an additive model formula in the design formula of DESeq2 and edgeR. We identified 134 (with DESeq2) and 127 (with edgeR) miRNAs differentially released (FDR ≤ 0.01) by 22Rv1/DCT^R^ clones (Table S1) and 1 (with DESeq2) and 3 (with edgeR) miRNAs differentially released (FDR ≤ 0.01) by DU-145/DCT^R^ clones (Table S2). We then considered only the miRNAs identified by both algorithms which were 105 miRNAs for 22Rv1/DCT^R^ clones (44 more released miRNAs, 61 retained/less released miRNAs) and 1 more released miRNA for DU-145/DCT^R^ clones (Figure 4A). We then focused on miRNAs more released (red in Figure 4A) and we selected those whose average number of normalized reads (NR) in all 22Rv1/DCT^R^ (or DU-145/DCT^R^) clones or in the corresponding parental cells was greater than 100 (Figure 4A, box i). Among them, we selected those with a difference greater than 10 NR (∆ > 10) between the clone with the lowest value and the biological replicate of the parental cells with the highest value (Figure 4A, box ii). Thus, we identified 12 miRNAs for 22Rv1/DCT^R^ clones (Figure 4B) and 1 miRNA (miR-146a-5p) for DU-145/DCT^R^ clones (Figure 4C and Figure S2).

**Figure 5:**
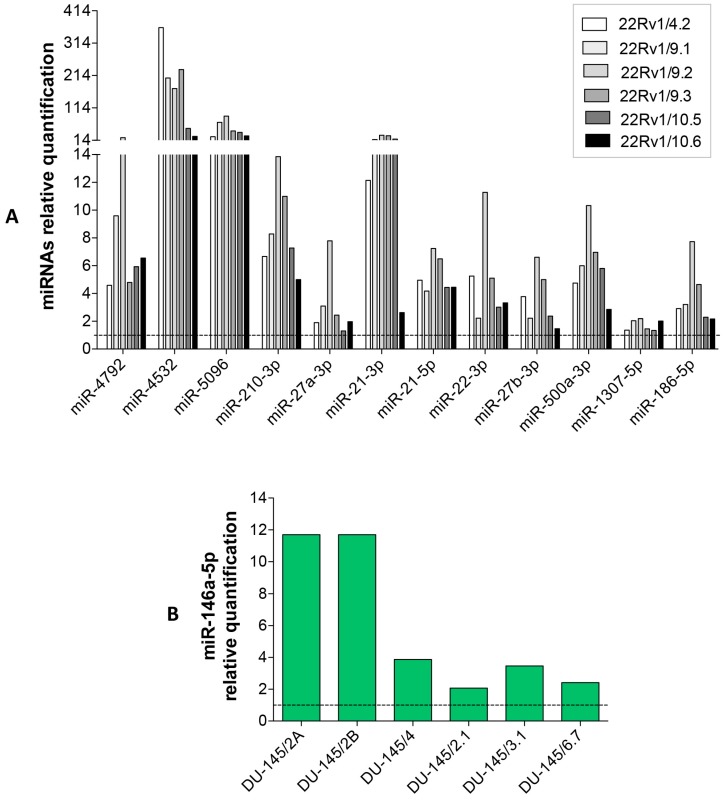
DCT^R^-miRNAs validation. Relative quantification with qRT-PCR of DCT^R^-miRNAs in the growth medium of 22Rv1/ (**A**) or DU-145/ (**B**) DCT^R^ clones with respect to parental cells (dotted line).

Finally, we validated the expression of these miRNAs (DCT^R^-miRNAs) in the growth medium of both 22Rv1/DCT^R^ and DU-145/DCT^R^ clones by qRT-PCR. With the exception of miR-1307-5p (whose level showed a slight change), the levels of all DCT^R^-miRNAs were higher in the growth medium of the DCT^R^ clones (Figure 5A,B) thus confirming the NGS data.

### 2.3. DCT^R^-miRNAs as Possible Biomarkers of DCT Resistance

To evaluate whether the level of the DCT^R^-miRNAs was affected by DCT, we measured by qRT-PCR the level of the 12 DCT^R^-miRNAs in the growth medium of parental cells (22Rv1 or DU-145) treated for three days with DCT and we calculated the fold change of these miRNAs in comparison to that found in the growth medium of untreated cells (column B, Table 1). Then, we evaluated the mean fold change of the DCT^R^-miRNAs (measured by qRT-PCR) in the growth medium of 22Rv1/DCT^R^ or DU-145/DCT^R^ clones with respect to that of the corresponding parental cells line (column A, Table 1). Finally, for each DCT^R^-miRNAs, we calculate the ratio between the fold change value of DCT-treated versus untreated cells and the mean fold change of DCT^R^-miRNA in 22Rv1/DCT^R^ or DU-145/DCT^R^ clones versus parental cells (column A/column B, Table 1). We observed that the level of 6 DCT^R^-miRNAs was similar in cells either resistant to or treated with DCT, whereas the level of the remaining 6 DCT^R^-miRNAs was higher (more than 2-fold) in cells resistant to DCT versus cells treated with DCT (Table 1, miRNAs underlined and in bold), suggesting that their differential release was specific of the DCT-resistant phenotype.

### 2.4. Expression Level of Selected DCT^R^-miRNAs in DCT^R^ Clones

**Figure 6:**
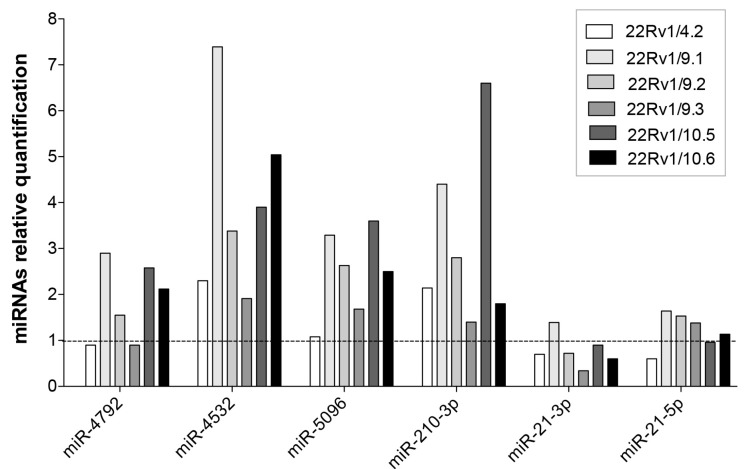
Intracellular level of selected DCT^R^-miRNAs in 22Rv1/DCT^R^ clones. Relative quantification by qRT-PCR of DCT^R^-miRNAs in the growth medium of 22Rv1/DCT^R^ clones with respect to parental cells (dotted line).

To gain more insight into the differential release of the DCT^R^-miRNAs by the DCT^R^ clones we measured the intracellular level of the DCT^R^-miRNAs specifically associated with DCT-resistant phenotype (Table 1, underlined and in bold) in 22Rv1/DCT^R^ clones with respect to parental cell lines (Figure 6). We found that DCT^R^-miRNAs were upregulated in 22Rv1/DCT^R^ clones (particularly miR-4532, miR-5096 and miR-210-3p) except for miR-21-3p and miR-21-5p whose level did not change significantly, suggesting that the release of DCT^R^-miRNAs by DCT^R^ PCa cells could be mediated by both active or passive mechanisms.

## 3. Discussion

Docetaxel (DCT) is the first line chemotherapy for patients who become insensitive to androgen deprivation therapy (castration-resistant prostate cancer, CRPC). Unfortunately, the DCT therapy frequently favors the development of DCT resistance in metastatic CRPC patients [2]. Hence, the discovery of biomarkers that indicate in advance the onset of DCT resistance could allow the early switch to other effective treatment options such as abiraterone acetate and enzalutamide [10,11]. While the detection of circulating miRNAs (c-miRNAs) in tumor patients is widely applied in clinical research [12,13,14], the detection of c-miRNAs in cancer patients under medical treatment is under-investigated, especially in PCa patients as confirmed by the poor availability of published data. So far, the change of miR-21 [5] and miR-210 [6] levels in the blood of PCa patients under DCT treatment have been associated with the clinical outcome. In addition, miR-141 [15,16], miR-146b-3p and miR-194 [16] have been shown to predict PCa clinical progression after therapies. Finally, in a recent work Lin et al. [17,18] demonstrated that miR-200a, miR-200b, miR-200c, miR-132, miR-375, miR-429 were associated with overall survival after DCT treatment.

In this work, we detected the miRNAs released by clones of 22Rv1 and DU-145 PCa cells resistant to DCT with the aim to identify those preferentially released by the DCT-resistant cells. The decision to isolate DCT^R^ clones from the androgen-dependent (22Rv1) and androgen-independent (DU-145) cell lines was due to the fact that DCT-based chemotherapy has become a therapeutic option not only for CRPC patients but also for those who have not yet reached the full androgen-independence [19]. In line with other data [20,21], we showed that the main molecular mechanism at the basis of the resistance of the DCT^R^ clones was the increase in the activity of the ABC membrane transporters, a family of ATP-dependent membrane-bound drug efflux pump that protects the tumor cells by cytotoxic drugs. In addition, we showed that both 22Rv1/DCT^R^ and DU-145/DCT^R^ clones had a generation time longer than those of the parental cell line. These data were in line with those obtained by Corcoran et al. [22]. We cannot exclude that the reduced growth rate of the DCT^R^ clones may contribute to the DCT-resistant phenotype.

Once the DCT^R^ clones were obtained, we detected miRNAs differentially released in the growth medium and selected those that passed several filters. First, we considered the miRNAs differentially released by all DCT^R^ clones derived from each cell line and this increased the probability that they could be those really released by DCT-resistant cells. Second, we focused on miRNAs that were more released and on these miRNAs we imposed a cut-off on normalized reads (NR > 100) as we believe that this level was adequate for an easier detection. Third, we selected miRNAs whose level were significantly different between DCT^R^ clones and parental cells by selecting not only the miRNAs more released by a |log2FC| > 1, but also the miRNAs whose level between the DCT^R^ clone with the lower NR and the parental line replicate with the highest NR was greater than 10 (∆ > 10). At the end, we selected 12 DCT^R^-miRNAs and 1 DCT^R^-miRNA released by 22Rv1/DCT^R^ and DU-145/DCT^R^ clones respectively. In our opinion, the low abundance of DU-145/DCT^R^-miRNAs in comparison with 22Rv1/DCT^R^-miRNAs depends on the fact that DU-145/DCT^R^ clones are more heterogeneous than 22Rv1/DCT^R^ clones as suggested by the molecular data (*BLC2* and *βIII tubulin* expression levels) and the different levels of *MDR1* activation. Another observation is that 22Rv1/DCT^R^ and DU-145/DCT^R^ clones did not release the same miRNAs. This finding is not completely surprising given that DCT^R^ clones derived from different tumor contexts and therefore the resistant phenotype may be different. As DCT resistance in PCa patients may arise from different tumor context as well, this observation underlines the importance of using more PCa cell lines to have a higher chance of identifying miRNAs which could potentially predict DCT resistance.

We then examined whether DCT^R^-miRNAs could be potential biomarkers of DCT resistance. In particular, we evaluated whether the release of the DCT^R^-miRNAs was affected also by the DCT treatment. We obtained promising results from the comparison of the DCT^R^-miRNAs released in the growth medium by DCT^R^ clones and by parental cells treated with DCT for 3 days. We showed that the release of miR-4792, miR-4532, miR-5096, miR-210-3p, miR-21-3p and miR-21-5p were higher in the growth medium of DCT^R^ clones than in DCT-treated cells thus rendering these miRNAs specific of DCT-resistant cells and then promising putative biomarkers of DCT resistance.

To be a good biomarker, a miRNA should be highly released before the onset of the DCT resistance. So far, many c-miRNAs diagnostic/prognostic of PCa have been identified, but it is still unclear if these c-miRNAs are useful in the management of PCa patients under DCT therapy. At the moment, only two c-miRNAs (miR-21, miR-210) have been associated to the outcome of DCT therapy [5,6] and, as such, have characteristics of predicting the DCT resistance of PCa patients. It has been shown that miR-21 level was elevated in CRPC patients, especially in those who developed resistance after DCT chemotherapy [5]. In the same way, it was demonstrated that the serum levels of miR-210 was higher in patients whose disease was resistant to DCT treatment as assessed by change in prostate specific antigen (PSA) [6]. It is worth noticing that both miR-21 and miR-210 belong to the 6 DCT^R^-miRNAs that were highly released only by cells resistant to DCT, suggesting that our approach to isolate DCT^R^ PCa cells and to select the differentially released miRNAs not affected by DCT treatment could represent a manner to discover miRNAs to be tested as predictive biomarkers of the DCT resistance. As a consequence, the other DCT^R^-miRNAs specifically released by DCT^R^ cells (miR-4792, miR-4532, miR-5096), and not yet tested in PCa patients, represent promising candidates to be tested as DCT predictive biomarkers in PCa patients.

Finally, we considered the intracellular level of the 6 DCT^R^-miRNAs in DCT^R^ clones to gain more insight on the miRNA release mechanism. We found that not all DCT^R^-miRNAs were upregulated in all 22Rv1/DCT^R^ clones suggesting that the release of these miRNAs by DCT-resistant PCa cells may be also mediated by an active mechanism. There are several reports in favor of either a specific or non-specific secretion of miRNAs by the cells (for review see [23,24]).

Among the miRNAs that were more upregulated (i.e., miR-210-3p, miR-4532 and miR-5096), miR-210 has been found to be transcriptionally activated by the hypoxia-inducible factor 1 α (HIF-1α) [25]. In particular, Cheng et al. [6] demonstrated that the treatment of PCa cells with hypoxic conditions determined the upregulation and the release of miR-210 in the growth medium. In addition, they demonstrated that high miR-210 serum level is associated with therapy (hormone therapy in combination with chemotherapy) resistance in CRPC patients. Given that it is known that tumor hypoxia is associated with therapy resistance [26], the author suggested that an increased hypoxia response signaling is present in a subset of CRPC patients, leading to increased miR-210 level and therapy resistance. So, the upregulation/release of miR-210-3p by 22Rv1/DCT^R^ clones is in line with these data: we can only speculate that an increased hypoxia signaling could be associated with DCT resistance mechanism of 22Rv1/DCT^R^ clones. However, miR-210 has a central role in promoting cancer-associated fibroblast (CAF) formation in PCa [27], hence in cancer progression, suggesting that it may act as a signal molecule in tumor microenvironment. Regarding miR-5096 and miR-4532, since they have been recently discovered, there are no reports on their biological functions. However, there are two studies that demonstrated that the transfer of miR-5096 through the gap junctions from glioma cells to astrocyte [28] or to human microvascular endothelial cells [29] promotes the glioma invasion with an unknown mechanism suggesting an oncogenic role of this miRNA, in line with its upregulation in 22Rv1/DCT^R^ clones.

In conclusion, we showed that DCT resistant PCa cells release in the growth medium miRNAs whose level change in PCa patients resistant to DCT therapy, suggesting that clones resistant to DCT represent a good in vitro model to identify potential DCT predictive miRNAs to be tested as c-miRNA in PCa patients during DCT treatment. In addition, we showed that not all miRNA specifically released by DCT^R^ clones were upregulated, suggesting that the miRNAs release may be mediated also by an active mechanism. In perspective, this in vitro approach for discovering miRNAs predictive of the onset of drug resistance may be applied to other tumor types as well as other anticancer drugs.

## 4. Materials and Methods

### 4.1. Cells and Culture Conditions

DU-145 and 22Rv1 cell lines were grown in RPMI 1640 medium added of 10% fetal bovine serum, 1% penicillin/streptomycin 2 mM and 1% l-glutammine 2 mM (Euroclone, Milan, Italy). Cells were incubated at 37 °C in a humidified atmosphere containing 6% CO_2_. For DCT treatment, cells were grown for 24 h and treated with DCT (Taxotere, 20 mg/mL, Sanofi Aventis, Milan, Italy) for 3 days.

### 4.2. Dose Response Curves

2 × 10^5^ cells were seeded per well (6 wells multiplate) and exposed to increasing concentration of DCT (Taxotere, 20 mg/mL, Sanofi Aventis). At specified time points, cells were fixed in 2% paraformaldehyde in PBS (Oxoid, Altrincham, Cheshire, UK), and subsequently stained with 0.1% crystal violet (SIGMA, St. Louis, MO, USA) dissolved in 20% methanol (SIGMA) and let dry at room temperature. Cells were then lysed with 10% acetic acid and the optical density (OD 590 nm) of the solution, detected with ChroMate Microtetraplate Reader apparatus (Awareness Technology, Westport, CT, USA), was used to measure cell proliferation.

### 4.3. Isolation and Characterization of DCT-Resistant (DCT^R^) Clones

To obtain DCT^R^ clones, 5 × 10^5^ cells/100 mm diameter dish were grown in presence of DCT of either 3 nM (22Rv1) or 4 nM (DU-145). Each 3–4 days, the medium was removed and replaced with fresh selective medium until colonies formation (30–50 days). Thereafter, independent colonies were isolated and expanded to clonal populations. To verify whether clones were still resistant to DCT, cells were seeded at density of 2 × 10^4^ cells/cm^2^ and 24 h later exposed at increasing concentrations of DCT (range 1–20 nM). After 72 h exposure, cell proliferation was determined as reported above. The generation times were calculated dividing the hours in culture by the number of cell doublings calculated as [lg_2_ (final cell number/initial cell number)/lg_2_ 2].

### 4.4. Rhodamine 123 Exclusion Assay

The assay was performed as previously described [30] with some modifications. Briefly, for each sample of DU-145 or 22Rv1 cells and DCT^R^ clones, 2 aliquots of 4 × 10^4^ cells/mL were incubated at 37 °C with 1 μL of Rhodamine 123 (Rh123) (1 mM, SIGMA) and with or without 1 μL of Verapamil (50 mM, SIGMA). After 30 min, cells were harvested and centrifuged and each aliquot was split into two aliquots and analyzed with FACScalibur cytofluorimeter (BD Biosciences, San Jose, CA, USA) immediately (aliquot 1) and after 1 h (aliquot 2). Rh123 green was recorded on FL1 channel and the gate was set to exclude fluorescence of both aliquot 1 samples and verapamil minus aliquot 2 sample. The Rh123 extrusion was detected in the verapamil plus aliquot 2 sample and expressed as percentage of cells with a fluorescence different from the one excluded from the gate.

### 4.5. Total RNA Isolation

Intracellular total RNA was extracted with miRNeasy mini kit (Qiagen, Hilden, Germany) following the manufacturer’s instructions. For NGS experiment, extracellular total RNA was isolated from 2 mL growth medium with QIAamp circulating nucleic acid kit (Qiagen), following the manufacturer’s instructions. For qRT-PCR analysis, extracellular total RNA extraction was performed as previously described [7].

### 4.6. miRNAs and mRNA Quantification

miRNAs and mRNAs quantification were performed as previously described [7]. Transcripts values were normalized to those obtained from the amplification of the internal control (GAPDH, HPRT1, β-actin for mRNAs and U6, sno-44, sno-55, sno-110 for intracellular miRNA and cel-miR-39 for extracellular miRNAs).

### 4.7. miRNA Profiling with Next-Generation Sequencing (NGS) Technology (Small RNA-Seq)

The small RNA libraries were constructed using TruSeq Small RNA kit (Illumina, San Diego, CA, USA) according to the manufacturer’s suggestions. cDNA libraries were loaded at six-plex level of multiplexing (~4 million reads per samples) into a flow cell V3, and sequenced in a single-reads mode (50 bp) on a MiSeq sequencer (Illumina). Raw reads were analyzed as described in Barsanti et al. [31]. Briefly, raw sequences were de-multiplexed using the Illumina pipline CASAVA v.1.8.2. software (Illumina). FastQC was used for quality check and primary reads were trimmed of adapters sequence using Cutadapt v1.2.1 [32]. Remaining reads, with a minimum length of 17 bzp and maximum 35 bp after trimming, were clustered for unique hits and mapped to pre-miRNA sequences present into the miRBase (rel. 21) employing miRExpress tool v 2.1.3 [33].

### 4.8. Statistical Analysis

Data are expressed as mean ± SD of at least three independent experiments, and analyzed with Student’s *t*-test.

The differential analysis was performed using two different statistical methods implemented in the two packages, edgeR [34] and DESeq2 [35] of the R Bioconductor repository. Exact *p*-values were adjusted for multiple testing according to Benjamini–Hochberg procedure. In order to control the batch effect of the two sequencing runs of the DU-145/DCT^R^ clones experiment, the analysis was corrected for baseline differences between the batches using an additive model in the design formula of both edgeR and DESeq2 approaches.

The hierarchical clustering was performed using the mean centered Log2 normalized reads, the Euclidean distance and complete agglomerative method.

## Figures and Tables

**Table 1:** DCT^R^-miRNAs level in the growth medium of DCT^R^ clones or DCT-treated cells.

DCT^R^/miRNAs	Column A *. DCT^R^-miRNAs Level in DCT^R^ Clones Versus Parental Cells Growth Medium	Column B **. DCT^R^-miRNAs Level in DCT-Treated Versus Untreated Cells Growth Medium	Column A/Column B	Unpaired *t*-Test Pvalue (A Versus B)
**miR-4792** ***	**8.94 ± 2.75**	**1.69 ± 0.11**	**5.27**	***p* < 0.05**
**miR-4532**	**175.20 ± 50.66**	**2.10 ± 0.48**	**83.42**	***p* < 0.01**
**miR-5096**	**48.61 ± 10.00**	**1.28 ± 0.03**	**37.97**	***p* < 0.001**
**miR-210-3p**	**8.80 ± 1.13**	**2.28 ± 0.19**	**3.86**	***p* < 0.001**
miR-27a-3p	3.48 ± 0.85	2.13 ± 0.2	1.63	NS
**miR-21-3p**	**17.7 ± 4.12**	**3.9 ± 0.49**	**4.53**	***p* < 0.01**
**miR-21-5p**	**5.18 ± 0.43**	**2.49 ± 0.39**	**2.07**	***p* < 0.001**
miR-22-3p	5.22 ± 1.21	3.86 ± 0.32	1.35	NS
miR-27b-3p	3.52 ± 0.77	4.29 ± 0.25	0.82	NS
miR-500a-3p	6.11 ± 1.01	3.78 ± 0.03	1.61	*p* < 0.05
miR-186-5p	3.82 ± 0.86	3.64 ± 0.43	1.05	NS
miR-146a-5p ****	5.87 ± 1.86	10.21 ± 1.46	0.57	NS

* Each value represents the mean ± SD of the DCT^R^/miRNAs level in the DCT^R^ clones. ** Each value represents the mean ± SD of the DCT^R^/miRNAs level in six biological replicates. *** DCT^R^-miRNAs whose column A/column B value was higher than 2 are underlined and in bold. **** miR-146a-5p was the only miRNA selected from DU-145/DCT^R^ clones. NS, not significant.

## Supplementary Materials

Supplementary materials can be found at www.mdpi.com/1422-0067/18/7/1512/s1.
