# Supplementary material for: The Prostate Cancer Cells Resistant to Docetaxel as in vitro Model for Discovering MicroRNAs Predictive of the Onset of Docetaxel Resistance
Source: Int J Mol Sci. 2017 Jul 13;18(7):1512. doi: 10.3390/ijms18071512 (PMC5536002; doi:10.3390/ijms18071512)
Supplement: Supplementary file 1 [file ijms-18-01512-s001.zip › Supplementary Figures.pdf]

Supplementary Materials: The Prostate Cancer Cells Resistant to Docetaxel as in vitro Model for Discovering MicroRNAs Predictive of the Onset of Docetaxel Resistance

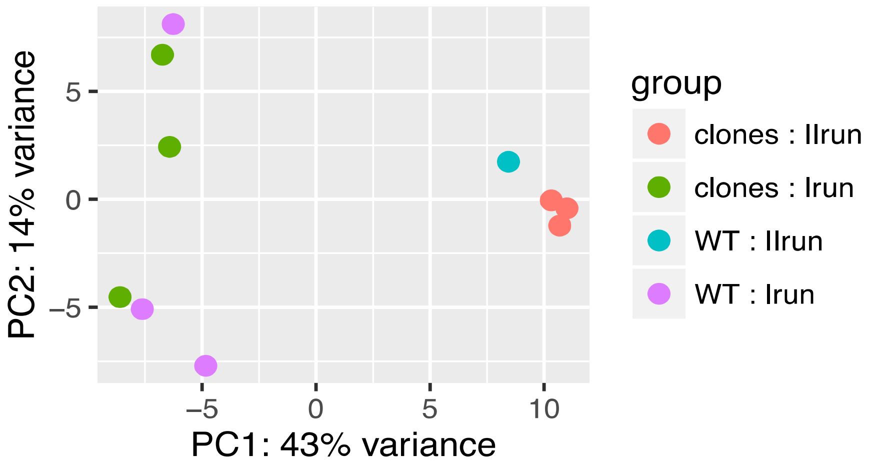

Figure S1. Principal component analysis (PCA) of DU-145/DCT[R] clones sequencing experiment.

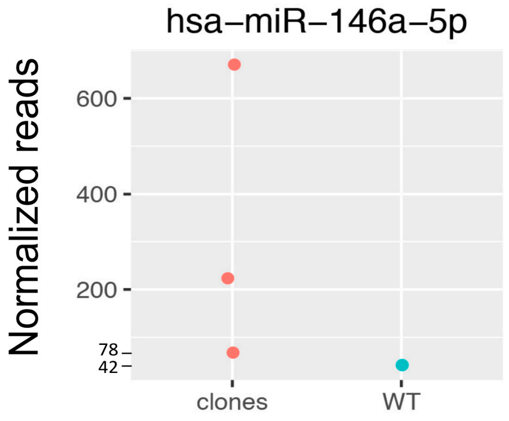

Figure S2. Normalized reads (NR) of miR-146a-5p in DU-145/DCT[R] clones (red spots) and parental cells biological replicates (blue spots) of DU-145/2.1, /3.1 and /6.7 clones.

Table S1–S2. List of differentially released miRNAs in 22Rv1 (**Table S1**) or DU-145 (**Table S2**) cells between DCT[R] clones and the correspondent parental cell lines identified by using edgeR and DESeq2 methods and using |log2FC|>1 and padj≤0.01 as criteria for selection. For each miRNA, base means across samples, log2 fold changes, standard errors, test statistics, p-values and adjusted p-values are reported.
